# Bio-augmentation and bio-stimulation with kenaf core enhanced bacterial enzyme activities during bio-degradation of petroleum hydrocarbon in polluted soil

**DOI:** 10.1038/s41598-023-50882-y

**Published:** 2024-01-02

**Authors:** Emmanuel Chukwuma Omenna, Kingsley Omage, Emmanuel Ezaka, Marshall Arebojie Azeke

**Affiliations:** 1 Institute of Agricultural Research and Training, Obafemi Awolowo University, Ile-Ife, Nigeria; 2 Division of Nephrology and Hypertension, Department of Medicine, Oregon Health and Science University, Portland, OR USA; 3 Department of Biochemistry, Faculty of Life Sciences, Ambrose Alli University, Ekpoma, Edo State Nigeria

**Keywords:** Environmental impact, Environmental biotechnology

## Abstract

Indigenous micro-organisms often possess the ability to degrade petroleum hydrocarbon (PHC) in polluted soil. However, this process can be improved by supplementing with nutrients or the addition of more potent microbes. In this study, the ability of kenaf-core to stimulate the PHC degradation capability of microbial isolates from PHC polluted soil samples was evaluated. The standard experimental methods used in this study include: the digestion and analysis of the physico-chemical properties of petroleum hydrocarbon contaminated and non-contaminated soil samples; evaluation of petroleum hydrocarbon biodegradation using bio-augmentation and bio-stimulation (with kenaf-core) treatments; and, determination of soil microbial enzyme activities. Results from this study show that K, Na, total nitrogen, organic carbon, exchangeable cations, and heavy metals were found to be significantly (*P* < 0.05) higher in the polluted soil than in the non-polluted soil. Also, the polluted samples had pH values ranging from 5.5 to 6.0 while the non-polluted samples had a pH of 7.6. The microbial enzyme activities were comparatively lower in the polluted soils as compared to the non-polluted soil. The percentage degradation in the kenaf-core treated samples (AZ_1_T_2_—78.38; BN_3_T_2_—70.69; OL_1_T_2_—71.06; OT_1_T_2_—70.10) were significantly (*P* < 0.05) higher than those of the untreated (AZ_1_T_1_—13.50; BN_3_T_1_—12.50; OL_1_T_1_—10.55; OT_1_T_1_—9.50). The degradation of petroleum hydrocarbon in the bio-augmented and bio-stimulated treatments increased with increasing time of incubation, and were higher than that of the untreated sample. Comparatively, the treatment with a combination of kenaf-core and rhamnolipid exhibited a significantly (*P* < 0.05) higher degradation rate than that of the treatment with only kenaf core or rhamnolipid. While, the bio-stimulated and bio-augmented treatments had appreciable microbial counts that are higher than that of the untreated. In conclusion, the nutrient-supplement with kenaf-core significantly enhanced microbial growth and activities in the soil, thus improving their ability to biodegrade petroleum hydrocarbons in the polluted soils. Thus, supplementing with Kenaf core to encourage microbiological degradation of petroleum hydrocarbon is recommended.

## Introduction

Petroleum hydrocarbon pollution has deleterious impact on soil health and this reduces soil enzyme activities which are indicators of the corresponding toxic effect of the pollutants on the soil ecosystems. Deterioration of soil due to oil spill and other xenobiotics may influence the activities of soil enzymes and thereby affect the health of the soil. The activities of these soil enzymes like catalase, amylase, urease, dehydrogenase, and alkaline phosphatase, are very sensitive to both natural and human (anthropogenic) disturbances, and they show rapid responses to these induced changes, like crude-oil pollution^1^. These changes can also affect the activities of the indigenous soil micro-organisms by reducing their activities. Certain bacteria isolated from crude-oil polluted soils have been reported to be efficient in the biodegradation process of crude oil-pollutants^2^. This is due to their ability to metabolize natural and industrially-produced petroleum-hydrocarbons^3^. They can produce bio-surfactants which could sometimes initiate the degradation of petroleum-hydrocarbons^4^. This degradation process or self-cleaning of oil spill may take much time^5^ due to low microbial population-density and activity in the site of pollution^6,7^. Although, petroleum-hydrocarbons are good sources of carbon and energy for the micro-organisms that can metabolise them, but they are inadequate because they do not contain sufficient amounts of other nutrients, such as nitrogen and phosphorus, which are required for the survival and growth of micro-organisms^8^. Also, a substantial spill usually overwhelms the natural supply of these nutrients in the ecosystem, and the microbial-degradation quickly becomes nutrient limited. However, with bio-augmentation or bio-stimulation, the ability of the indigenous micro-organisms to degrade the petroleum hydrocarbon pollution can be enhanced.

In the process of bio-stimulation, the microbes are enriched with nutrients which in turn enhances their ability to degrade the petroleum hydrocarbon pollutants, thus improving the bioremediation process. Nutrients addition can stimulate petroleum-hydrocarbon decomposition by enhancing microbial population and their metabolic activities. The survival and growth of indigenous or inoculated oil-degrading microbes are facilitated by the addition of nutrients or other growth-limiting co-substrates. Thus, bioremediation can be enhanced by bio-augmentation with potent microbes as well as bio-stimulation with mineral-nutrients and/or surfactants^9^. The use of straw or plant material such as kenaf core powder, which acts as an absorbent and/or biosurfactants to clean oiled surfaces, and the addition of this material to encourage microbiological degradation of oil have been recommended^10^. Kenaf plant is a low-risk cash crop which demands little application of chemicals. It grows fast and absorbs carbon dioxide gas which helps in the reduction of global warming. It has also gained wide usage as an absorbent. The fibre from kenaf plant is non-abrasive, and has been reported to be more effective than the classical remediates like silica and clay^10^. Kenaf core is a non-toxic material which encourages microbiological decomposition of petroleum hydrocarbon.

The use of non-toxic, non-abrasive kenaf (*Hibiscus cannabinus*) bast and core fibers have been reported to be effective in bioremediation^11^. However, there are dearth of information in the literature describing the effectiveness of the locally produced kenaf core fibre (Ifeken-100) in the bioremediation of crude-oil polluted soils, especially within the Niger Delta or Southern region of Nigeria. This study was therefore carried out in an attempt to give a scientific rationale for the use of locally produced kenaf core fibre (Ifeken-100) in the bioremediation of crude-oil polluted soil in Sothern Nigeria. In this study, we assessed the possibility of the application of locally produced kenaf core fibre (Ifeken-100) as a nutrient stimulant for petroleum hydrocarbon degrading micro-organisms in polluted soil samples from the Southern region of Nigeria. The effectiveness of the locally produced kenaf core in the bioremediation process was compared with that of a natural surfactant, rhamnolipid, which is commercially available. This in vitro study also evaluated the physico-chemical properties, soil microbial enzyme activities and microbial count of the petroleum hydrocarbon polluted and non-polluted soil samples, as well as the petroleum hydrocarbon degradation capability of the microbial isolates from the different soil samples.

## Materials and methods

### Kenaf sample collection and preparation

The indigenous variety of kenaf crop (Ifeken-100) was used for this study. The kenaf plant was grown in the experimental field of the Institute of Agricultural Research and Training, Ibadan. Prior to the harvesting and use of the kenaf crop for the experiment, it’s taxonomy (kenaf crop-Ifeken-100) was identified at the Department of Botany, Ambrose Alli University, Ekpoma, Edo State, Nigeria. The collection and experimental studies on the kenaf crop were also in compliance with the relevant institutional, national, and international guidelines and legislation. The plant was harvested at ten-weeks-after- planting (10WAP). The stem was retted, properly washed, separated and sundried. The dried cores were pulverized into powder (0.20 mm) using hammer mill and stored until use at room temperature.

### Description of site and collection of soil samples

The soil samples used in this experiment were collected from oil polluted and non-polluted sites in three states, located within the Southern region of Nigeria. To increase the precision of sample collection, the random soil sampling method was employed. The different locations from which the soil samples were collected are namely: Bayelsa State (Otukpoti and Azikoro) which lie within the same latitude 04.84^o^N and longitude 06.27°E; Edo State (Ologbo—which lies within latitude 06.06°N and longitude 05.66°E and Benin—which lies within latitude 06.35^o^N; and longitude of 05.63°E); non-polluted soil sample was collected from Moor Plantation, Ibadan, Oyo State—which lies within latitude 7.39°N and longitude 3.5°E. The crude-oil contaminated soil samples were collected from petroleum polluted sites (from 0–20 cm depth) at two locations each from Bayelsa and Edo State. The soil samples were stored and conveyed to the laboratory in ice-bags.

### Physico-chemical analysis of petroleum hydrocarbon contaminated soil samples

Prior to the physico-chemical analyses, the soil samples were air dried and sieved through a 2 mm Lab-Sieve. The soil solution was prepared by mixing it with distilled water, in the ratio of 1:2. The pH and temperature of the soil solution were determined using Jenway pH metre (Model SP701) in accordance with the manufacturer’s directions. Particle size analysis was conducted using the Hydrometer method and the organic matter was determined by chromic acid oxidation method, as prescribed by Umanu and Nwachukwu^12^ with slight modifications according to Ikoro et al.^13^. Exchangeable bases were determined by the neutral ammonium acetate saturation.

### Soil samples digestion

Prior to the determination of the soil’s macronutrients, the soil samples were digested. About 0.2 g of each soil sample was measured into a Kjeldhal digestion tube. One selenium tablet was added followed by 4 ml of concentrated Tetra-oxosulphate (VI) solution. The tube and its content were placed in the digestion block and allowed to digest for 5 h. The digest was allowed to cool, after which it was rinsed and transferred into a 100 ml volumetric flask and subsequently made up to the mark with distilled water. The digest was subjected to analysis for sodium and potassium, using the flame photometer, while calcium and magnesium were determined using the atomic absorption spectrophotometer (AAS).

### Microbial isolation and screening from the crude-oil polluted soils

The medium used, mineral-salt-medium (MSM), was prepared by measuring 0.27 g of KH_2_PO_4,_ 1.4 g of Na_2_HPO_4,_ 0.8 g of NaCl, and 0.2 g of KCl, and dissolved in one litre of deionized water, after which the mixture was autoclaved at 121 °C for 15 min^14^. The isolation of the oil-degrading microorganisms using MSM was in accordance with the protocol described by Churchill et al.^15^. Ten grams of each sieved soil sample was added to 100 ml of the MSM and incubated in a shaker at 30 °C and 140 rpm and allowed to stay for 48 h. About 1 ml of the broth culture was transferred into 50 ml freshly prepared MSM containing 2% crude oil, and incubated under the same condition. The MSM used for the isolation contained 2% crude oil (PHC) as source of carbon and energy, and was done using the spread plate method. The plates were incubated at 30 °C for 72 h. The efficient oil-degrading microbes isolated from the crude-oil polluted soils were *Paenalcaligenes suwonesis, Lactobacillus fermentum, Lactobacillus fermentum,* and *Lactobacillus nagelii*, as we previously reported^2^. The colonies that were morphologically different were isolated and sub-cultured on nutrient agar, and the process was repeated until a pure culture was obtained. The microbial-growth was monitored through optical-density and the absorption was measured using a spectrophotometer at a wavelength of 620 nm^16^.

### Experimental design for in-vitro-degradation study

Crude oil biodegradation evaluation was performed in 250 ml Erlenmeyer flasks having 50 ml of mineral salt medium (MSM) and 2% crude oil. Sterilized MSM was inoculated with 2 ml each of the isolates (*Paenalcaligenes suwonesis, Lactobacillus fermentum, Lactobacillus fermentum,* and *Lactobacillus nagelii*) from the Azikoro (AZ1), Benin (BN3), Ologbo (OL1) and Otukpoti (OT1), then supplemented with 3% (w/v) kenaf core and/or rhamnolipid at 35 °C on a rotary shaker Incubator (Stuart Orbital Model S1500, Japan) at 180 rpm. The experiment was conducted as follows:

- MSM + Isolate + Crude oil, coded as “T_1_”
- MSM + Isolate + Kenaf Core + Crude oil, coded as “T_2_”
- MSM + Kenaf Core + Crude oil, coded as “T_3_”
- MSM + Kenaf Core + Rhamnolipid + Crude oil, coded as “T_4_”
- MSM + Rhamnolipid + Crude oil, coded as “T_5_”
- MSM + Isolate + Rhamnolipid + Crude oil, coded as “T_6_” (positive control)
- MSM + Crude oil, coded as “T_7_” (negative control)

All treatments were conducted in triplicates. MSM consist of 0.27 g of KH_2_PO_4,_ 1.4 g of Na_2_HPO_4,_ 0.8 g of NaCl, and 0.2 g of KCl in one litre of distilled water. T_3,_ T_4_, T_5_ and T_7_ were used for bio-stimulation study while T_1_, T_2_ and T_6_ were tagged as to reflect the place of origin of the isolates and were used for the bio-augmentation study as described below:

- *Bio-augmentation treatments*: AZIT_1_; AZ1T_2_; AZ1T_6_; BN3T_1_; BN3T_2_; BN3T_6_; OL1T_1_; OL1T_2_; OL1T_6_; OT1T_1_; OT1T_2_; OT1T_6_.
- *Bio-stimulated treatments*: T_3_; T_4_; T_5_; T_7_.

### Quantitative evaluation of petroleum hydrocarbon (PHC) bio-degradation

About 2.0 ml each from the four PHC-degrading isolates were poured into a 500 ml round bottom flask containing 100 ml of sterile defined MSM with 2% crude oil and then supplemented with 3% rhamnolipid and/or 3% kenaf core as shown in the design above. The treatments with isolates were known as bio-augmentation and those without isolates were known as bio-stimulation. All the treatments were incubated for 90 days at 30 °C and 200 rpm. The concentration of residual petroleum hydrocarbon and pH as well as microbial count was monitored. The total petroleum hydrocarbon was determined in all treatments at the interval of 15, 30, 45, 60, 75, and 90 days of incubation using the modified spectrophotometric method^17^. About 5 ml of each sample was taken from all the treatments and were mixed properly with an equivalent quantity of dichloromethane (Cl_2_CH_2_) at a ratio of 1:1 to extract the hydrocarbons from each treated sample. The extracted hydrocarbons from the samples were then determined using a Cecil Spectrophotometer (Model CE1010, UK) at a wavelength of 600 nm. To determine the residual amounts of hydrocarbons in all the treatments, a standard curve was also prepared using known amount of crude-oil^17^. The percentage of hydrocarbon degradation was determined by calculating the difference between the initial and final concentration of crude-oil in each treatment using the formula:

$$ \% {\text{PHC}}\;{\text{degradation}} = \frac{Initial\; conc - Final\; conc}{{Initial \;conc}} \times { 1}00 $$

### The pH of media supplemented with kenaf core and/or rhamnolipid

The pH of all the treatments was determined at ambient temperature using glass electrode, (Jenway pH meter; Model no: S1500). A MSM consisting of kenaf core, rhamnolipid, crude oil and various inorganic salts were dissolved in 1000 dm^−3^ of distilled H_2_O^18^. About 100 ml of MSM was dispensed into different round bottom flasks, 3% of kenaf core and/or 3% rhamnolipid were added into the designated flasks for bio-stimulated treatments and the solution was sterilized by autoclaving. About 2 ml of the 3 h broth culture (peptone broth) of the selected organism was seeded into the flask designated as bio-augmented treatments.

### Determination of soil microbial-enzyme activities

The activities of enzymes such as amylase, dehydrogenase, urease and phosphatase in each crude oil polluted and non-polluted soil samples were assayed according to standard methods, with slight modifications where necessary, as described by Margesin and Schinner^19^. The detailed methodology for each enzyme is presented below;

### Determination of soil-microbial amylase activity

5 g of air-dried soil was weighed into 100 ml conical flask. 1.5 ml of toluene was added to the dry soil sample in the flask. The mixture was shaken and allowed to stand for 15 min at room temperature. 10 ml of distilled water were added followed by 5 ml of 2% solution of soluble starch. They were incubated at 37 °C for 5 h. Afterwards, 10 ml of the suspension was centrifuged at 3000 rpm for 20 min. The spectrophotometric-absorbance was read at 660 nm wavelength.

### Determination of soil microbial-dehydrogenase activity

The dehydrogenase activity was evaluated using the protocol prescribed by Tabatabai^20^. About 1 g of sieved soil was placed in test tubes, mixed with 1 ml of 3% dilute 2, 3, 5-triphenyl tetrazolium chloride (w/v) and stirred with a glass rod. After 96 h of incubation at 27 °C, 10 ml of ethanol was added to each test tube and vortexed for 30 s. The tubes containing the mixtures were incubated for 1 h to allow the suspended soil particles to sediment. The resultant supernatant (about 5 ml) was carefully transferred into test tubes with Pasteur’s pipettes. The formazan’s spectrophotometric-absorbance was read and recorded at the wavelength of 485 nm. Dehydrogenase converts 2, 3, 5-triphenyl tetrazolium chloride to formazan. The concentration of formazan that was formed was evaluated.

### Determination of soil microbial-phosphatase activity

This was determined by weighing 0.1 g of air-dried soil into a 50 ml conical flask, with addition of 4 ml buffer (pH 6.5) solution. This was followed by addition of 0.25 ml of toluene and 1 ml of 0.115 M p-nitrophenyl phosphate solution. The flask was swirled for 30 s and was then incubated at 37 °C for 1 h. After incubation, 1 ml of 0.5 M sodium hydroxide solution was introduced to the mixture. The soil suspension was then filtered using a what-man filter paper. The optical density of the filtrate was measured at 430 nm using spectrophotometer.

### Determination of soil microbial-urease activity

One gram of fresh soil was weighed into a 100 ml volumetric flask, followed by 1 ml of toluene and the solution was allowed to stand for 15 min. Afterwards, 10 ml of buffer (pH 7) and 5 ml of 10% urea solution were added. The mixture was incubated at 37 °C for 3 h. After incubation, the volume of the mixture was made up to the mark with distilled water. The mixture was filtered using a Watman filter paper and then 0.5 ml of the filtrate was taken into 25 ml volumetric flask. This was followed by addition of 5 ml distilled water, 2 ml phenolate solution and 1.5 ml sodium hypo-chloride solution. The volume of the mixture was increased to 25 ml with distilled water and the optical density was measured at 630 nm.

### Statistical analysis

The data obtained were subjected to analysis of variance (ANOVA). Comparison of Mean was made using a least significant difference at the *P* < 0.05 probability level by the Statistical Analysis System (SAS) program (SAS Institute, Cary, N.C.).

### Ethics approval

The ethical approval for this research was obtained from the Research Ethics Committee of the Department of Biochemistry, Ambrose Alli University, Ekpoma Nigeria.

### Ethical responsibilities of authors

All authors have read, understood, and have complied as applicable with the statement on "Ethical responsibilities of Authors" as found in the Instructions for Authors and are aware that with minor exceptions, no changes can be made to authorship once the paper is submitted.

## Results

### Physico-chemical properties of the crude oil-polluted and non-polluted soil

**Figure 1:**
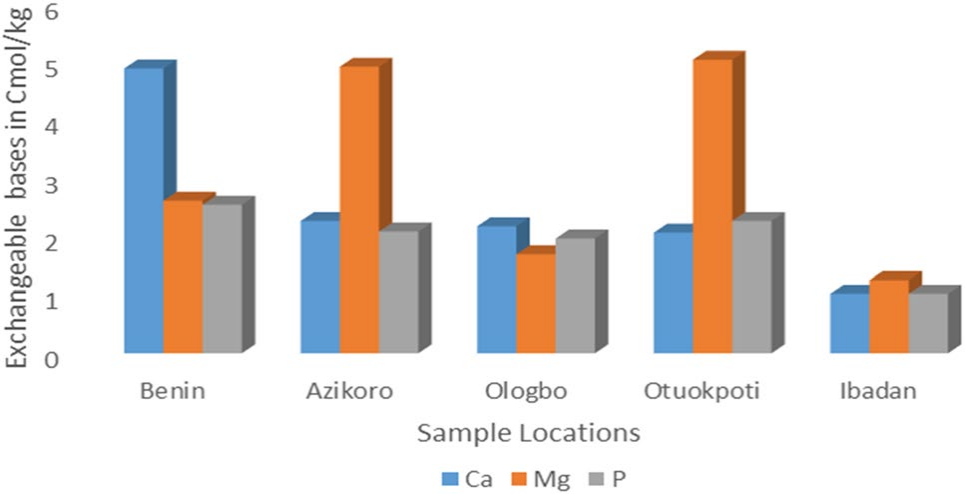
The macro-nutrient content of the crude-oil polluted and non-polluted soil from different locations.

**Figure 2:**
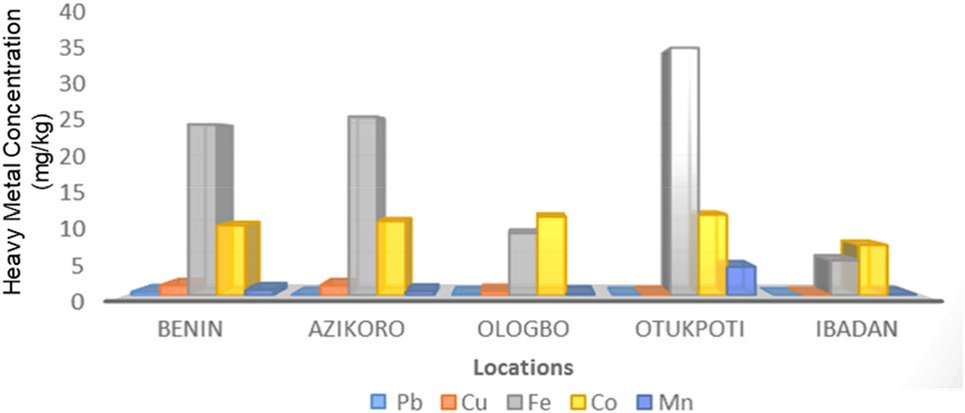
The elemental composition of the crude-oil polluted and non-polluted soils.

The results presented in Table 1 show the physical and chemical properties of the polluted and non-polluted soil samples. The evaluation of the physical texture show that all the soil samples were sandy-loamy soil with > 50% sand content, except Otukpoti soil sample which was clayey-loamy with 28.76% sand. All the polluted soil samples have pH values ranging from 5.5 to 6.0 while the non-polluted (Ibadan) soil has a pH value of 7.6. The implication is that crude oil spillage makes the soil lightly acidic. Other physico-chemical parameters of the soil like K, Na, total nitrogen, organic carbon and exchangeable cations were significantly higher in polluted soil than in non-polluted soil as shown in Table 1 and Fig. 1. Among the polluted soil samples, Ca^2+^ is reportedly highest in the Benin sample, while Mg^2+^ is comparably higher in Azikoro and Otukpoti samples. Heavy metals were significantly higher in all the crude-oil polluted soils than the non-polluted Ibadan soil (Fig. 2). However, among the crude oil-polluted soil samples, iron (Fe) content in the sample from Otukpoti is comparably the highest.

**Table 1:** Physico-chemical properties of crude-oil polluted and non-polluted soil from different locations.

Soil location	% Sand	% Clay	% Silt	pH	K (ppm)	Na (ppm)	% O.C	% T.N	H+	Texture
Benin (Polluted)	58.76^c^	9.4^b^	31.84^b^	5.8^b^	6240^a^	881.16^a^	2.58^c^	0.26^b^	0.12^b^	Sandy-loamy
Azikoro (Polluted)	76.76^b^	7.4^c^	15.84^c^	5.8^b^	6080^b^	844.45^b^	2.50^d^	0.25^c^	0.12^b^	Sandy-Loamy
Ologbo (Polluted)	78.76^b^	9.4^b^	11.84^d^	6.0^c^	6080^b^	826.09^c^	2.67^b^	0.27^a^	0.11^c^	Sandy-Loamy
Otukpoti (Polluted)	28.76^d^	33.4^a^	37.84^a^	5.5^b^	6240^a^	826.09^c^	2.70^a^	0.27^a^	0.14^a^	Clayey-loamy
Ibadan (non-polluted)	92.20^a^	3.4^d^	4.40^e^	7.6^a^	1500^c^	300.00^d^	1.68^e^	0.14^d^	0.02^d^	Sandy-loamy

Data represent Mean values of triplicate determinations (n = 3): Mean with different letter superscripts (^a, b, c, d, e^) in a column are significantly (*P* < 0.05) different. O.C = Organic Carbon; T.N = Total Nitrogen; ppm = part per million.

### Activities of soil microbial enzymes from different soil samples

The activities of enzymes in the soil samples which determines the rate of biochemical reactions and soil health are as indicated in Table 2. The result showed enzyme activities in the petroleum hydrocarbon polluted soils in relation to the non-polluted soil sample as well as its importance in soil nutrients circulation. Generally, the activities of the enzymes were shown to be comparatively lower in the polluted soil samples as compared to the non-polluted soil. The least amylase and phosphatase activities were recorded in the soil sample from Azikoro, while the least dehydrogenase activity was recorded in the sample from Benin. However, among the polluted soil samples, the sample from Azikoro exhibited the highest urease activity.

**Table 2:** Enzyme activities in oil polluted and non-polluted soils.

Soil sample	Amylase (μg glucose/g/soil)	Phosphatase (μg P-nitrophenol/g/soil)	Dehydrogenase (μg formazan/g/soil)	Urease (μg urea/g/soil)
Azikoro	17.90 ± 0.20^e^	10.40 ± 0.20^e^	1.40 ± 0.01^c^	3.03 ± 0.01^b^
Otukpoti	18.50 ± 0.01^d^	13.08 ± 0.04^c^	1.72 ± 0.03^b^	1.98 ± 0.02^d^
Ologbo	20.60 ± 0.12^c^	13.60 ± 0.03^b^	1.20 ± 0.01^d^	2.02 ± 0.01^c^
Benin	25.00 ± 0.10^b^	12.80 ± 0.01^d^	1.09 ± 0.02^e^	1.48 ± 0.20^e^
Ibadan	32.10 ± 0.02^a^	15.00 ± 0.10^a^	3.10 ± 0.03^a^	5.11 ± 0.01^a^

Data represent Mean ± SD values of triplicate determinations (n = 3): Mean with different letter superscripts (^a, b, c, d, e^) in a column are significantly (*P* < 0.05) different.

### Degradation of petroleum hydrocarbons in the soils from the different locations

In Tables 3, 4, 5 and 6, the treatment of crude oil-contaminated soil samples from Azikoro, Benin, Ologbo and Otukpoti, with kenaf core shows a steady increase in the percentage degradation of the petroleum hydrocarbon content, from the 15th day to the 90th day of in vitro incubation. In all, the percentage degradation in the kenaf core-treated samples (AZ_1_T_2_; BN_3_T_2_; OL_1_T_2_; OT_1_T_2_) are significantly (*P* < 0.05) higher than those of the untreated samples (AZ_1_T_1_; BN_3_T_1_; OL_1_T_1_; OT_1_T_1_). However, the rhamnolipid-treated samples (AZ_1_T_6_; BN_3_T_6_; OL_1_T_6_; OT_1_T_6_) exhibited a significantly (*P* < 0.05) higher percentage degradation as compared to the kenaf core-treated and untreated samples.

**Table 3:** Percentage degradation of petroleum-hydrocarbons by Azikoro-1 isolate at *in-vitro* incubation.

Treatment with Azikoro^1^	15th Day	30th Day	45th Day	60th Day	75th Day	90th Day
AZ_1_T_1_	3.00^c^	3.45^c^	3.30^c^	6.68^c^	10.01^c^	13.50^c^
AZ_1_T_2_	21.90^b^	38.11^b^	37.67^b^	60.00^b^	72.03^b^	78.38^b^
AZ_1_T_6_	30.00^a^	59.15^a^	58.00^a^	75.44^a^	80.70^a^	95.48^a^

Data represent Mean values of triplicate determinations (n = 3): Mean with different letter superscripts (^a, b, c^) in a column are significantly (*P* < 0.05) different; T_1_ = MSM + Azikoro^1^isolate + crude oil; T_2_ = MSM + Azikoro^1^isolate + kenaf core + crude oil; T_6_ = MSM + rhamnolipid + Azikoro^1^isolate + crude oil (positive control).

**Table 4:** Percentage degradation of petroleum-hydrocarbons by Benin-3 isolate at *in-vitro* incubation.

Treatment with Benin^3^	15th Day	30th Day	45th Day	60th Day	75th Day	90th Day
BN_3_T_1_	2.25^c^	3.30^c^	3.00^c^	6.00^c^	9.41^c^	12.50^c^
BN_3_T_2_	20.10^b^	25.00^b^	24.53^b^	50.10^b^	69.00^b^	70.69^b^
BN_3_T_6_	29.00^a^	57.85^a^	56.00^b^	79.45^a^	80.70^a^	91.00^a^

Data represent Mean values of triplicate determinations (n = 3): Mean with different letter superscripts (^a, b, c^) in a column are significantly (*P* < 0.05) different; T_1_ = MSM + Benin-3 isolate + crude oil; T_2_ = MSM + Benin-3 isolate + kenaf core + crude oil; T_6_ = MSM_+_ rhamnolipid + Benin-3 isolate + crude oil (positive control).

**Table 5:** Percentage degradation of petroleum-hydrocarbons by Ologbo-1 isolate at *in-vitro* incubation.

Treatment with Ologbo^1^	15th Day	30th Day	45th Day	60th Day	75th Day	90th Day
OL_1_T_1_	2.50^c^	3.52^c^	3.45^c^	6.01^c^	9.50^c^	10.55^c^
OL_1_T_2_	20.15^b^	26.50^b^	25.36^b^	51.00^b^	70.10^b^	71.06^b^
OL_1_T_6_	29.50^a^	59.50^a^	58.10^a^	79.85^a^	81.50^a^	91.65^a^

Data represent Mean values of triplicate determinations (n = 3): Mean with different letter superscripts (^a, b, c^) in a column are significantly (*P* < 0.05) different; T_1_ = MSM + Ologbo^1^ isolate + crude oil; T_2_ = MSM + Ologbo^1^ isolate + kenaf core + crude oil; T_6_ = MSM_+_ rhamnolipid + Ologbo^1^ isolate + crude oil (positive control).

**Table 6:** Percentage degradation of petroleum-hydrocarbons by Otukpoti-1 isolate at *in-vitro* incubation.

Treatment with Otukpoti^1^	15th Day	30th Day	45th Day	60th Day	75th Day	90th Day
OT_1_T_1_	2.50^c^	3.52^c^	3.45^c^	6.01^c^	9.50^c^	10.55^c^
OT_1_T_2_	20.15^b^	26.50^b^	25.36^b^	51.00^b^	70.10^b^	71.06^b^
OT_1_T_6_	29.50^a^	59.50^a^	58.10^a^	79.85^a^	81.50^a^	91.65^a^

Data represent Mean values of triplicate determinations (n = 3): Mean with different letter superscripts (^a, b, c^) in a column are significantly (*P* < 0.05) different; T_1_ = MSM + Otukpotio^1^ isolate + crude oil; T_2_ = MSM + Otukpotio^1^ isolate + kenaf core + crude oil; T_6_ = MSM + rhamnolipid + Otukpotio^1^ isolate + crude oil (positive control).

### Comparative degradation of petroleum hydrocarbons in bio-stimulated and bio-augmented treatments

**Figure 3:**
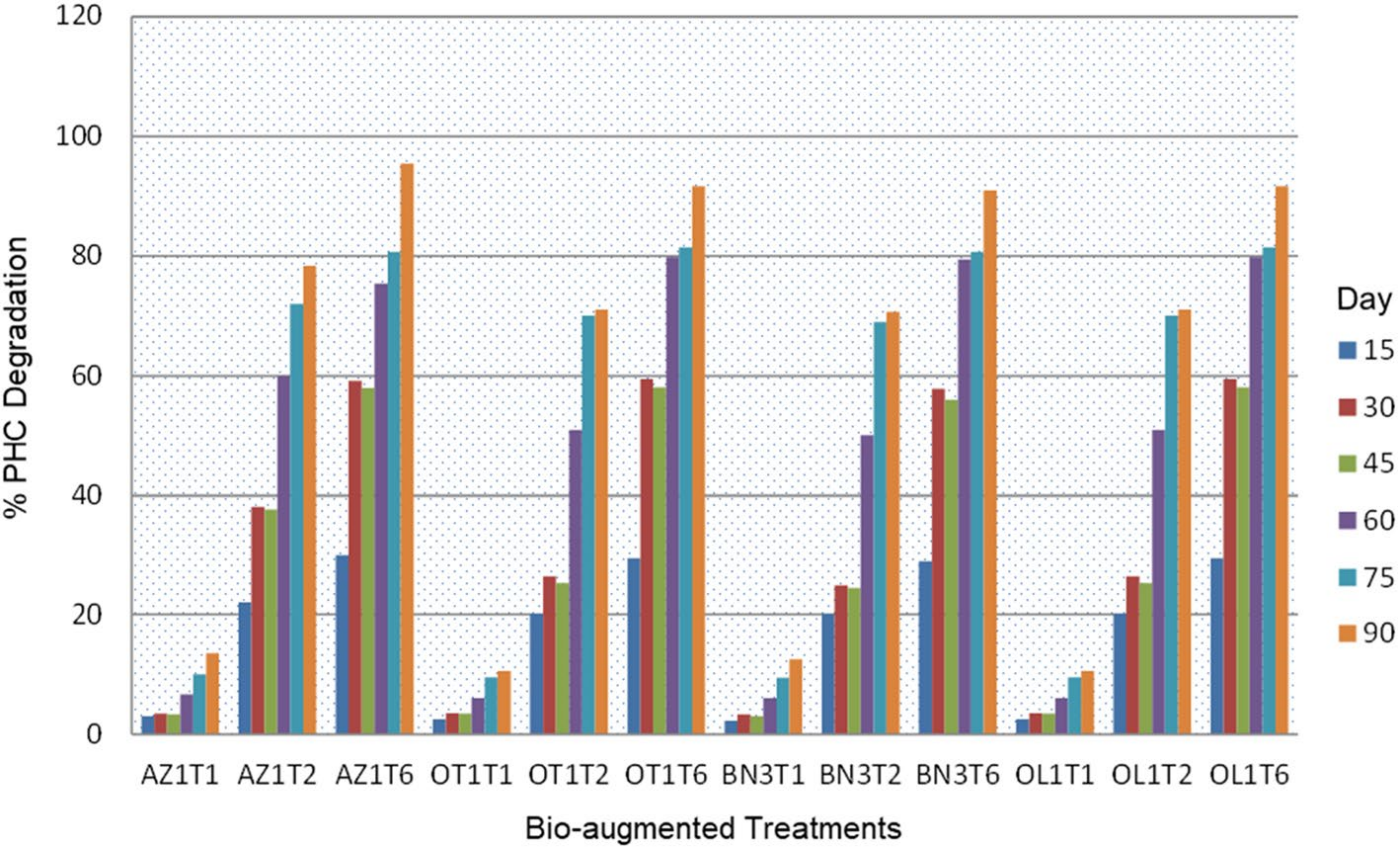
Comparative degradation of petroleum-hydrocarbons in bio-augmented treatments. Data represent Mean values of triplicate determinations (n = 3): T_1_ = MSM + Isolate + Crude oil; T_2_ = MSM + Isolate + Kenaf core + Crude oil; T_6_ = MSM + Rhamnolipid + Isolate + crude oil (positive control).

In Table 7, the degradation of petroleum hydrocarbon in the bio-stimulated treatments increased with increasing time of incubation. Comparatively, the treatment with a combination of kenaf core and rhamnolipid (T_4_) exhibited a significantly (*P* < 0.05) higher degradation rate than that of the treatment with only kenaf core (T_3_) or only rhamnolipid (T_5_). However, treatment with kenaf core (T_3_) resulted in a significantly (*P* < 0.05) higher degradation rate than that of the untreated (T_7_). This trend is also true for the bio-augmented treatments in Fig. 3. Here, the degradation activities of the microbial isolates from the different soil samples were enhanced by the addition of kenaf core.

**Table 7:** Comparative degradation of petroleum-hydrocarbon in bio-stimulated treatments.

Treatment	15th day	30th day	45th day	60th day	75th day	90th day
T_3_	16.29^c^	25.23^c^	34.30^c^	55.80^c^	70.10^c^	76.00^b^
T_4_	28.50^b^	55.05^b^	60.10^b^	71.40^b^	78.32^b^	82.57^b^
T_5_	25.40^a^	50.00^a^	56.72^a^	65.02^a^	70.80^a^	76.00^a^
T_7_	ND	ND	ND	ND	1.02^d^	2.1^c^

Data represent Mean values of triplicate determinations (n = 3): Mean with different letter superscripts (^a, b, c, d^) in a column are significantly (*P* < 0.05) different; T_3_ = MSM + kenaf core + crude oil; T_4_ = MSM + kenaf core + rhamnolipid + crude oil; T_5_ = MSM + rhamnolipid + crude oil; T_7_ = MSM + crude oil (negative control = -ve). ND = Nothing detected.

### Comparative bacteria growth at *in-vitro* degradation of petroleum hydrocarbon in soil samples from the different locations

**Figure 4:**
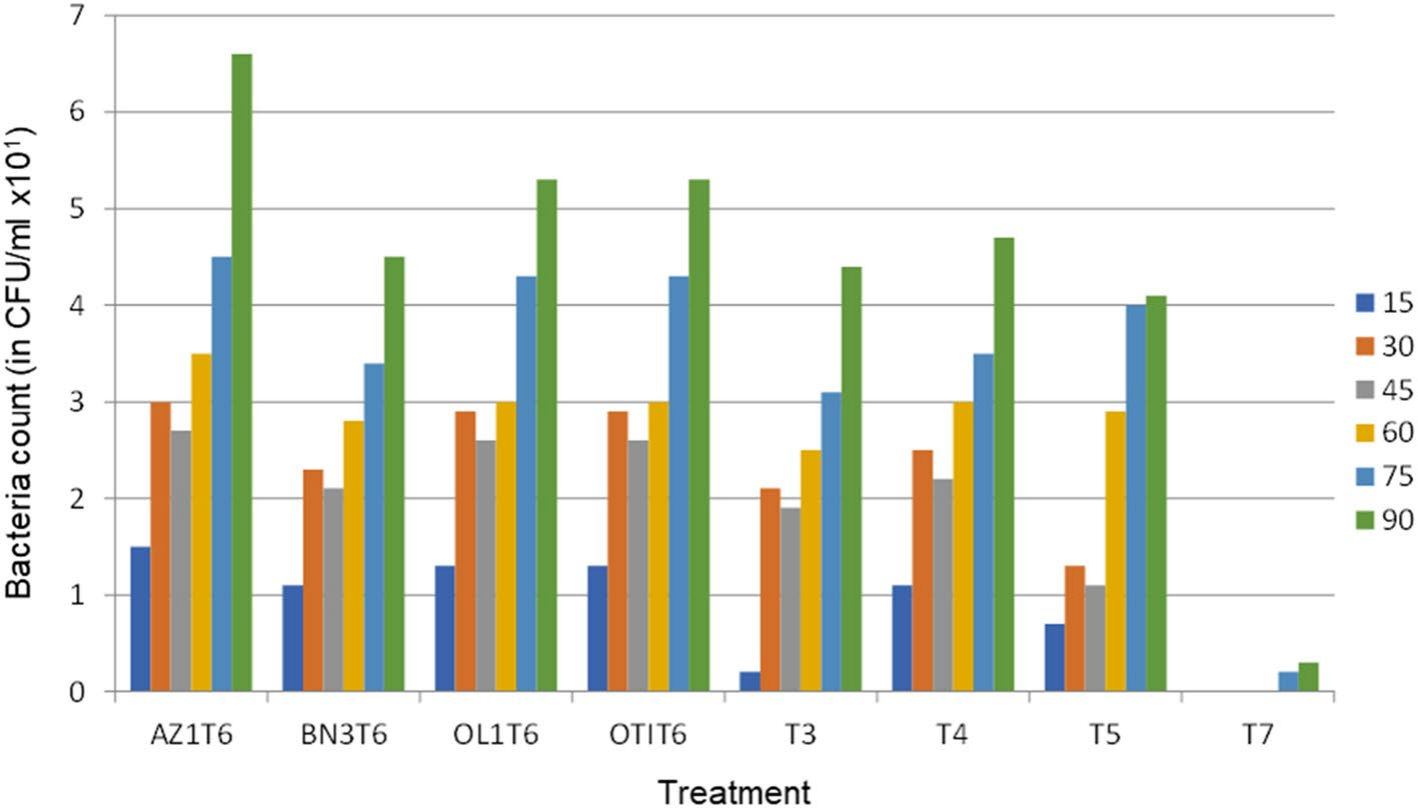
Comparative bacteria count at *in-vitro* degradation of PHC. Data represent Mean values of triplicate determinations (n = 3): T_6_ = MSM_+_ rhamnolipid + named bacteria + crude oil (positive control); T_3_ = MSM + kenaf core + crude oil; T_4_ = MSM + kenaf core + rhamnolipid + crude oil; T_5_ = MSM + rhamnolipid + crude oil; T_7_ = MSM + crude oil (negative control = −ve).

In our previous study, we reported that *Paenalcaligenes suwonesis, Lactobacillus fermentum, Lactobacillus fermentum,* and *Lactobacillus nagelii*, were the efficient oil-degraders isolated from the crude-oil polluted soils under study^2^. Presently, we show that in Table 8 and Fig. 4, the growth patterns of all the isolates followed the same trend. The nutrients synergy between kenaf core and rhamnolipid significantly influenced the bacteria growth. The result of the bacteria counts revealed that both bio-stimulated and bio-augmented treatments had appreciable microbial counts, but there was no microbial growth at the first 75 day of incubation for T_7_ (negative control). Interestingly, the AZ_1_T_6_ (MSM + rhamnolipid + Azikoro isolate) treatment showed an exceptionally rapid microbial growth, which was significantly (*P* < 0.05) different from others even among the bio-augmented treatments. All the treatments, apart from the negative control (T_7,_) had similar microbial growth pattern with the same lag phase at first six weeks. Comparatively, the bacteria count in the soil sample from Azikoro (AZ_1_T_6_) is significantly (*P* < 0.05) higher than those from the other locations, while the soil sample from Benin (BN_3_T_6_) location exhibited the least count.

**Table 8:** Bacteria count (in CFU/ml × 10^1^) at *in-vitro* degradation of PHC.

Treatment	15th Day	30th Day	45th Day	60th Day	75th Day	90th Day
AZ1T1	0.3^e^	0.6^h^	0.5^g^	0.9^e^	1.1^g^	1.4^g^
AZ1T2	1.2^b^	2.2^c^	2.0^c^	3.0^d^	4.2^c^	5.0^c^
AZ1T6	1.5^a^	3.0^a^	2.7^a^	3.5^a^	4.5^a^	6.6^a^
BN3T1	0.2^e^	0.5^h^	0.4^g^	0.7f	0.9^h^	1.1^h^
BN3T2	1.1^c^	1.5f	1.4^e^	3.0^b^	3.9^d^	4.8^d^
BN3T6	1.1^c^	2.3^c^	2.1^b^	2.8^c^	3.4^e^	4.5^e^
T3	0.2^e^	2.1^d^	1.9^c^	2.5^d^	3.1f	4.4^e^
OL1T1	0.3^e^	0.5^h^	0.4^g^	0.8^e^	1.0^g^	1.3^g^
OL1T2	1.1^c^	1.9^e^	1.7^d^	2.5^d^	3.0f	4.0f
OL1T6	1.3^b^	2.9^a^	2.6^a^	3.0^b^	4.3^b^	5.3^b^
T4	1.1^c^	2.5^b^	2.2^b^	3.0^b^	3.5^e^	4.7^d^
OT1T1	0.3^e^	0.5^h^	0.4^g^	0.8^e^	1.0^g^	1.3^g^
OT1T2	1.1^c^	1.9^e^	1.7^d^	2.5^d^	3.0f	4.0f
OT1T6	1.3^b^	2.9^a^	2.6^a^	3.0^b^	4.3^b^	5.3^b^
T5	0.7^d^	1.3^g^	1.1f	2.9^b^	4.0^d^	4.1f
T7	ND	ND	ND	ND	0.2^i^	0.3^i^

Data represent Mean values of triplicate determinations (n = 3): Mean with different letter superscripts (^a, b, c, d, e, f, g, h, i^) in a column are significantly (*P* < 0.05) different; T_1_ = MSM + named bacteria + crude oil; T_2_ = MSM + named bacteria + kenaf core + crude oil; T_3_ = MSM + kenaf core + crude oil; T_4_ = MSM + kenaf core + rhamnolipid + crude oil; T_5_ = MSM + rhamnolipid + crude oil; T_6_ = MSM_+_ rhamnolipid + named bacteria + crude oil (positive control); T_7_ = MSM + crude oil (control), ND = Nothing detected.

## Discussion

The physico-chemical analysis of the oil-polluted soil samples show that the texture, organic content, total nitrogen, potassium content, available phosphorous and the pH were significantly affected. This finding is in line with previous report by Wang et al.^21^ which stated that petroleum, which is rich in carbon, with traces of nitrogen in its compounds, can change the composition and structure of soil organic matter and impact the C/N, C/P, salinity, pH and conductivity of soil. The results of the elemental analysis show that crude oil polluted soils were significantly higher in lead, Cobalt, iron, manganese, and copper content than in non-polluted soil. The slightly acidic pH and significantly higher heavy metals, Mg^2+^, Ca^2+^, K, Na, total nitrogen, organic carbon and exchangeable cations in the polluted soil samples points at the toxic effects of the petroleum hydrocarbon on the soil ecosystem. This shows that there is a positive relationship between crude-oil contamination and metal accumulation in the affected environment. This observation is in line with the report by Adams et al.^22^ which stated that crude-oil contamination increased the level of some heavy-metals which can bio-accumulate and bio-magnify thereby causing adverse effects on the soil health. Heavy metals can be extremely toxic as they can block the functional groups of vital soil microbial enzymes^22^. In the same vein, it was observed that when soil is polluted, its physico-chemical properties are affected and this may decrease its productive potentials.

The total nitrogen and organic carbon obtained in crude-oil polluted soils from Niger Delta were significantly higher than non-polluted soil sample from Ibadan. In their study, Al-Mailem et al.^23^ reported that crude-oil polluted soil samples from different countries (Kuwait, Lebanon, Egypt, and Germany) had phosphorus content ranging from 0.3 to 1.3 mgkg^−1^ while potassium content ranged from 0.5 to 5.2 mg kg^−1^_._ In agreement with that, Liao et al.^24^ reported that petroleum-hydrocarbons can react with the inorganic-nitrogen and phosphorus present in the soil, thereby limiting the process of nitrification and removal of phosphoric acid. Thus, the effective nitrogen and phosphorus in the soil may be reduced and its absorption by the crops can be affected. Although, soil is said to be heterogeneous in nature and as such two soil samples from the same geographical location can show variability in their physico-chemical characteristics^25^. The pH values of the petroleum hydrocarbon-polluted soil samples (from Azikoro, Benin, Ologbo, and Otukpoti) which ranges from 5.50 to 6.80 (acidic) were significantly lower than that (pH 7.6—basic) obtained from the non-polluted Ibadan soil sample. This may also vary from one location to another, because, previous report by Al-Mailem et al*.*^23^ indicated that oil polluted sandy soil from Kuwait had a pH value of 6.5 while oil polluted loamy soil from Lebanon soil had a pH value of 7.1, and oil-polluted loamy soil from Germany had a pH value of 7.3. The disruption of the pH balance of the soil by petroleum hydrocarbon pollution often affect the enzymatic activities of the soil microbes which are pH-dependent. For example, majority of the heterotrophic microbes like fungi and bacteria thrive better in neutral or alkaline pH, while some fungi are tolerant to acidic-conditions. The survival and bioremediation potential of these bacteria, therefore, depends on the pH of the soil.

Soil enzymes have significant impacts on the general soil biology, particularly on growth and nutrient uptake by the plants in ecosystem. But, pollution such as oil spillage and accumulation of other xenobiotics can alter soil environmental indices^26^. The results of microbial enzyme activities demonstrated that the soil sample from Ibadan has the highest activity of amylase followed by that from Benin, Ologbo and Otukpoti, while the soil sample from Azikoro has the least amylase activity. Wyszkowska and Wyszkowski^27^ previously reported in their study that soil contaminated with petroleum substances had decreased amylase activity for the first few weeks. We also observed similar pattern in our present study, which indicate the adverse effect of the petroleum hydrocarbon on the amylase activity of the soil samples. Studies have shown that α-amylase is synthesised in plants, micro-organism and soils. Research evidence suggested that several enzymes participated in the hydrolysis of starch but of major importance is α-amylase, which converts starch-like-substrate to glucose or oligosaccharide for microbial uptake^28^. This is essential for soil microbial health and by extension, a better capacity to carry out bioremediation.

Our determination of the phosphatase activity in the crude-oil polluted and non-polluted soil samples demonstrated that Ibadan soil sample, which is not polluted, has a higher phosphatase activity than all polluted soil samples. Although, Ologbo and Otukpoti soils have a comparable phosphatase activity and these were significantly higher than that of other polluted soils. All the crude-oil-contaminated soil samples recorded significantly lower phosphatase activity than in non-polluted Ibadan soil. This observation is in line with the report from the research published by Wyszkowska and Kucharski^29^ which stressed that phosphatase enzyme was more resistant to petroleum-hydrocarbon-contaminants than other enzymes. Pertinently, phosphatase is an enzyme with great agronomic-relevance, because it catalyzes the hydrolysis and transformation of organic-phosphorus into different forms of inorganic-phosphorus which can subsequently be assimilated by plants^30^. Also, phosphatase plays vital role in enzymatic reaction that release phosphate ions into soil and this is essential in phosphorus cycling. They are broad groups of enzymes which are capable of catalysing the hydrolysis of esters, and phosphoric-acid anhydrides^31^. Although, many literatures reported that both phosphatase and urease were less commonly used in assessing the impacts of crude-oil pollution on the soil microbial-enzymes, it was also emphasized that they are involved in macronutrient cycles and can provide insight on the metabolic capacity of the soil^25,32^. In comparison with other enzymes, Wyszkowska and Kucharski^29^ reported that the phosphatases strongly resisted the pollutants more than other enzymes. It also exhibited weakest stimulation by the reclamation treatments. In the soil ecosystem, it was believed that this enzyme (phosphatase) plays critical roles in phosphorus-cycle but evidence showed that they were correlated with phosphorus-stress and plant growth^33^.

The results of other enzymatic parameters were significantly higher in the non-polluted (Ibadan) soil than other soil samples which were polluted. Our findings reveal that the dehydrogenase activity was highest in Ibadan soil followed by Otukpoti and Azikoro, whereas Benin soil sample had the least. The result from this study is strongly in support of the report by Gospodarek et al*.*^34^ which stated that the dehydrogenase activity showed fluctuations during the bio-remediation of soils polluted with petroleum-derived–substances (PDS). This was correlated with the microbial-respiration and degradation of crude-oil. In the same vein, Gospodarek et al*.*^34^ reported that an introduction of the bacterium, *Paracoccus* to the soil polluted with polycyclic-aromatic hydrocarbons (PAH) caused an increase in dehydrogenase activity (> 35%) after 28 days. In this study, the observed low dehydrogenase activity in crude oil polluted soils suggested there was a decrease in total soil microbial-respiration, since the level of dehydrogenase activity in the soil was a reflection of microbial population and respiration rate^35,36^. Likewise, Gianfreda et al.^37^ also observed that there was decline in the dehydrogenase activity and high levels of polycyclic aromatic hydrocarbons (PAHs) in the soil polluted over five decades with petroleum-hydrocarbons. The observation from this study partly disagrees with that from Ezirim et al.^26^ who emphasized that there was significantly higher dehydrogenase activity in petroleum-hydrocarbons-polluted soils than non-polluted soil. Lian et al.^38^ reported that hydrocarbons and organic acids in the soil serve as H donors for the micro-organisms and the enzyme substrate catalysed by dehydrogenase are oxidised by oxygen through the cytochrome system from which the released energy was the main energy source for the heterotrophic micro-organisms.

The level of oil pollution affects the quantitative and qualitative composition of soil micro-flora. Soil enzymes were closely-related with the amount of petroleum degrading micro-organisms participating directly or indirectly in degradation of petroleum contaminants. Lian et al.^38^ opined that dehydrogenase exhibited tolerance against high-concentration of petroleum-hydrocarbons pollution. Moreover, the data obtained from this study support the report by Lian et al.^38^ which stated that enzymatic reaction in the soil was higher in non-polluted soil than in petroleum-hydrocarbon-contaminated soils. Ezirim et al*.*^26^ pin-pointed that petroleum hydrocarbon-polluted top-soil has higher dehydrogenase activity of 3.43 μg than petroleum hydrocarbon-polluted sub-soil with 1.3 μ/g dehydrogenase activity. However, the data from this study and the reported data by Gospodarek et al*.*^34^ prove that there is significantly higher dehydrogenase activity in non-polluted soil than in petroleum hydrocarbon-polluted soil samples. Although, Gospodarek et al*.*^34^ reported that after four years of petroleum derived substance-contamination, dehydrogenase activity began to increase. The authors added that the increased dehydrogenase activity was because the micro-organisms were able to adapt to the pollutants and utilize them as sources of energy and carbon. Many researches have used dehydrogenase activity as an indicator for biological activities in the soil^33^. However, dehydrogenase enzyme is an integral part of intact-cells but does not accumulate extracellularly in the soil. Dehydrogenase catalyses the oxidation of soil organic-matter by transferring protons or electrons from substrate donors to acceptors. These processes are part of the respiration pathways of soil micro-organisms which are closely-related with the soil-types, soil-air, and soil–water conditions^30,39^. More so, Baran et al.^40^ and Dindar et al.^41^ suggested that there was increase in respiratory intensity as well as increase in enzyme activity, growth of micro-organisms and a gradual decomposition of pollutants after a period of stress. The results from this study are in support of the report by Futughe et al.^42^ which emphasized that dehydrogenase enzyme in the soil is very important as it gives an indication of the potential of the soil to support biochemical processes which are essential in maintaining soil fertility. In addition, the research study by Futughe et al.^42^ suggested that the water content and the temperature of the soil can indirectly influence the dehydrogenase activity and also affect the soil redox-status. In the same vein, Fiedler et al.^43^ highlighted that the relationship between dehydrogenase activity and redox potentials as well as Fe^2+^ was used to illustrate the reactions of soil micro-organisms to the changes in the soil environment. For instance, lack of oxygen may trigger facultative anaerobes to initiate metabolic process involving dehydrogenase activities and the use of Fe^3+^ as terminal electron-acceptor and this process can affect the availability of iron to plants in the ecosystem^33^. Some studies demonstrated that the reducing-conditions in soil was associated with high Fe^2+^ concentration in soil-solutions.

Our results also show that the urease activities in petroleum hydrocarbon-polluted soil samples were significantly lower than that of the non-polluted (Ibadan) soil, in the following decreasing order of urease activity; Ibadan > Azikoro > Ologbo > Otukpoti > Benin. This is in agreement with the results from the study reported by Gospodarek et al*.*^34^ which emphasized that there was reduction in urease activity due to the introduction of petroleum–derived substances into the soil. Though, Gospodarek et al*.*^34^ reported that nutrients addition has both stimulatory and inhibitory effects on the soil-enzymes, depending on the kind of petroleum-hydrocarbons and type of enzymes. Gospodarek et al*.*^34^ pointed out that there was fifty percentage decrease in urease activity in petroleum-derived substance contaminated soil than the control. However, the results from this study are not in agreement with the report by Efsun et al.^30^ which stated that urease activity was significantly higher in the soil contaminated with 5% crude-oil than the soil polluted with 0.5% crude-oil. From this study, we can infer that the level of crude-oil pollution was significantly higher in Azikoro and Ologbo soils than others. Soil urease activity is a good indicator of the mineralization potential of organic nitrogen compounds in soil^30^. In this study, all the enzyme activities are significantly lower in all crude oil polluted soils than the non-polluted soil. Although, the numerical data obtained from this present study were slightly lower than the reported data on hydrocarbon polluted soils from Egbema by Ezirim et al.^26^. However, this variability was earlier explained by Gospodarek et al*.*^34^ to be as result of soil enzymes and soil fauna occurrence or the type of petroleum.

Micro-organisms grow well where the rate of dissolution of hydrocarbons is slow because there is possibility of the presence of soluble substrates in the environment. But, when the microbial growth rate is faster than the rate of dissolution of hydrocarbons, then the biodegradation potential of the micro-organism is limited. The four isolates, *Paenalcaligenes suwonesis, Lactobacillus fermentum, Lactobacillus fermentum,* and *Lactobacillus nagelii*^2^, from Niger Delta namely, Azikoro-1, Benin-3, Otukpoti-1 and Ologbo-1 have demonstrated their capacities to utilize and degrade petroleum hydrocarbons within the first ninety days of *in-vitro* incubation. Similarly, the population count of Azikoro-1 (660 CFU/ml) was significantly higher than that of Ologbo-1 or Otukpoti-1(530 CFU/ml), while Benin-3 (480 CFU/ml) was the least at 90th day of incubation. The increase in microbial growth resulted in the increased petroleum hydrocarbon degradative potentials of the isolates. Azikoro-1 had the highest petroleum hydrocarbon degradation followed by Ologbo-1 and/or Otukpoti-1, while Benin-3 had the least petroleum hydrocarbon degradation.

Our results from the bio-stimulated and bio-augmented treatments showed that cultured bacteria strains can metabolize/degrade petroleum hydrocarbons. This observation supports the research findings reported by Heider et al*.*^3^ which emphasized that in the last decade of microbiological research, only few particular microorganisms were able to anaerobically-catabolize hydrocarbons. In addition, hydrocarbons like aliphatic-alkenes, and alkanes (with 6–20 carbon atoms), monocyclic-alkylbenzenes, (such as toluene, ethylbenzene, propylbenzene, p-cymene, xylene and ethyltoluene), and isomers like benzene and naphthalene can be anaerobically degraded. The rate of degradation of petroleum-hydrocarbon recorded in this study supports other literature reports which highlighted that some petroleum-hydrocarbons can be anaerobically degraded by pure bacteria cultures^3^. The isolates from this study may possibly be among the species of denitrifying, ferric-reducing, and sulfate-reducing-bacteria which can anaerobically utilize alkyl-benzene and other saturated hydrocarbons as substrates. Heider et al.^3^ stated categorically that bacteria-isolates with this metabolic capacity must have alternative oxygen-independent source for the initial attack on their hydrocarbon-substrates.

The bacteria numeration at *in-vitro* incubation revealed a gradual increase in the population of the micro-organisms in all the treatments except in the negative control (T_7_). There was no observed microbial growth in the negative control group for the first sixty days of incubation. However, we recorded a very negligible amount of microbial growth at the 75^th^ and 90^th^ day of incubation. Generally, we observed that the microbial growth in the treatments was significantly higher at the 90th day of incubation. The bio-augmented treatments show different amount of microbial growth which is in the descending order; AZ1T6 > OL1T6 > OT1T6 > AZ1T2. This observation is in agreement with the reported data by Mineki et al.^44^ which stated that there were enhanced microbial-growth and degradation efficiency when *Trichoderm / Hypocrea* was used in the degradation of polycyclic aromatic-hydrocarbons (PAHs), after 7 and 14 days of incubation. The results from this study partially agree with the reported data by Abdulsalam and Omale^45^ which stated that after the lag-phase in the microcosm bio-stimulation, there was accelerated microbial growth between week one and three, which resulted in a rapid degradation of the petroleum-hydrocarbon contaminants by 63.1%. In addition, micro-organisms played major roles in bioremediation and their absolute numbers can determine the overall degradation abilities^45^. In view of this result, Al-Hawash et al.^46^ earlier reported that the main factor affecting petroleum hydrocarbon degradation was the availability of micro-organisms which can catabolize the pollutants. The absence of microbial growth for the first sixty days observed in the negative control is at variance with report by Al-Hawash et al.^46^ which emphasized that microorganisms that use petroleum-hydrocarbons as foods were easily found in places exposed to crude-oil contamination such as: crude-oil spillage sites, shipping lanes, ports, oil-fields, gas-stations and others. However, our results agree with the report of Al- Hawash et al.^46^ which stated that petroleum hydrocarbon biodegradation efficiency was a function of growth factor such as nutrient accessibility, oxygen, temperature, cellular transport properties, chemical composition of the growth media and optimal enzyme condition.

The bio-stimulated treatments (T4, T3, and T5) have comparable numbers of micro-organisms with BN3T2, BN3T6, OL1T2, and OT1T2. This observation suggested that it may be possible for inocula to die after a period of time due to stress or pollution. More importantly, the results from this study show that kenaf-rhamnolipid supplemented treatment (T4) had better microbial growth than other bio-stimulated treatments. This result strongly supported the findings of Gospodarek et al.^34^ which stated that nutrients addition can have either stimulatory or inhibitory effect on the microbial activities for bioremediation. We also observed that the growth patterns of all the isolates followed the same trend and the nutrients synergy obtained from kenaf core and rhamnolipid had significantly influenced the bacteria growth. The result of bacteria counts reveal that the bio-stimulated and bio-augmented treatments had appreciable microbial counts but there was no microbial growth at the first 75 day of incubation for T_7_ (negative control). This could mean that with bio-stimulation, the indigenous micro-organisms were capable of dealing with the prevailing crude oil pollution. The microbes were enriched with nutrients and in turn got involved in the biodegradation of the pollutants. However, the MSM-rhamnolipid + Azikoro isolate (AZ1T6) showed an exceptionally-rapid microbial growth, which was significantly different from others, even among the bio-augmented treatments.

## Conclusion

This study shows that soil contaminated with petroleum hydrocarbons had elevated level of macro- and micro-elements, total organic and water-soluble carbons, as well as significantly lower amylase, phosphatase, dehydrogenase and urease activities. We report that locally produced kenaf core (Ifeken-100) is effective in the bioremediation of crude-oil polluted soils. Also, we demonstrated that the combination of the locally produced kenaf core and 95% rhamnolipid provided nutrients synergy to indigenous or native micro-organisms thereby acting as an effective bio-stimulant. Thus, the combination of kenaf core and rhamnolipid appears to be more promising in terms of hydrocarbon degradation than other stimulated treatments. This study therefore provides a scientific rationale for the possible use of locally produced kenaf core fibre (Ifeken-100) in the bioremediation of crude-oil polluted soil in Sothern Nigeria.

## Data Availability

The datasets used and/or analysed during the current study available from the corresponding author on reasonable request.
